# The complexities and challenges of preventing and treating nontuberculous mycobacterial diseases

**DOI:** 10.1371/journal.pntd.0007083

**Published:** 2019-02-14

**Authors:** Susan L. Baldwin, Sasha E. Larsen, Diane Ordway, Gail Cassell, Rhea N. Coler

**Affiliations:** 1 Infectious Disease Research Institute, Seattle, Washington, United States of America; 2 Department of Global Health, University of Washington, Seattle, Washington, United States of America; 3 Mycobacteria Research Laboratories, Department of Microbiology, Immunology and Pathology, Colorado State University, Fort Collins, Colorado, United States of America; 4 Department of Global Health and Social Medicine, Harvard Medical School, Boston, Massachusetts, United States of America; 5 PAI Life Sciences, Seattle, Washington, United States of America; Insitut Pasteur de Tunis, TUNISIA

## Abstract

Seemingly innocuous nontuberculous mycobacteria (NTM) species, classified by their slow or rapid growth rates, can cause a wide range of illnesses, from skin ulceration to severe pulmonary and disseminated disease. Despite their worldwide prevalence and significant disease burden, NTM do not garner the same financial or research focus as *Mycobacterium tuberculosis*. In this review, we outline the most abundant of over 170 NTM species and inadequacies of diagnostics and treatments and weigh the advantages and disadvantages of currently available in vivo animal models of NTM. In order to effectively combat this group of mycobacteria, more research focused on appropriate animal models of infection, screening of chemotherapeutic compounds, and development of anti-NTM vaccines and diagnostics is urgently needed.

**Key learning points**

- Pulmonary diseases due to NTM are an increasing global health concern.
- Prevalence of NTM is increasing in the US and has surpassed pulmonary tuberculosis (TB).
- Successful treatment for NTM infection is complicated by the large number of NTM species (>170), difficulties with diagnosis, and few therapeutic options once diagnosed.
- *Mycobacterium avium* complex (MAC) lung disease treatment is costly, lengthy, and often results in toxic side effects.
- New therapeutic strategies are urgently needed, including more efficacious drugs with fewer adverse side effects and novel treatment options that could reduce treatment time and toxicity.
- Prioritizing funding and research using NTM animal models to dissect NTM pathogenesis and host-directed responses and test novel therapeutic regimens against these infections may help combat these widespread pathogens.

**Top five papers**

1. Griffith DE, et al. An official ATS/IDSA statement: diagnosis, treatment, and prevention of nontuberculous mycobacterial diseases. *Am J Respir Crit Care Med*. 2007;175(4):367–416.
2. Haworth CS, et al. British Thoracic Society guidelines for the management of non-tuberculous mycobacterial pulmonary disease (NTM-PD). *Thorax*. 2017;72(Suppl 2):ii1-ii64.
3. Andrejak C, et al. Characterization of mouse models of *Mycobacterium avium* complex infection and evaluation of drug combinations. *Antimicrobial agents and chemotherapy*. 2015;59(4):2129–35.
4. Bryant J, et al. Emergence and spread of a human transmissible multidrug-resistant nontuberculous mycobacterium. *Science*. 2016;354:751.
5. Huh HJ, et al. Recent advances in molecular diagnostics and understanding mechanisms of drug resistance in nontuberculous mycobacterial diseases. Infection, Genetics and Evolution, https://doi.org/10.1016/j.meegid.2018.10.003.

## Introduction

**Fig 1:**
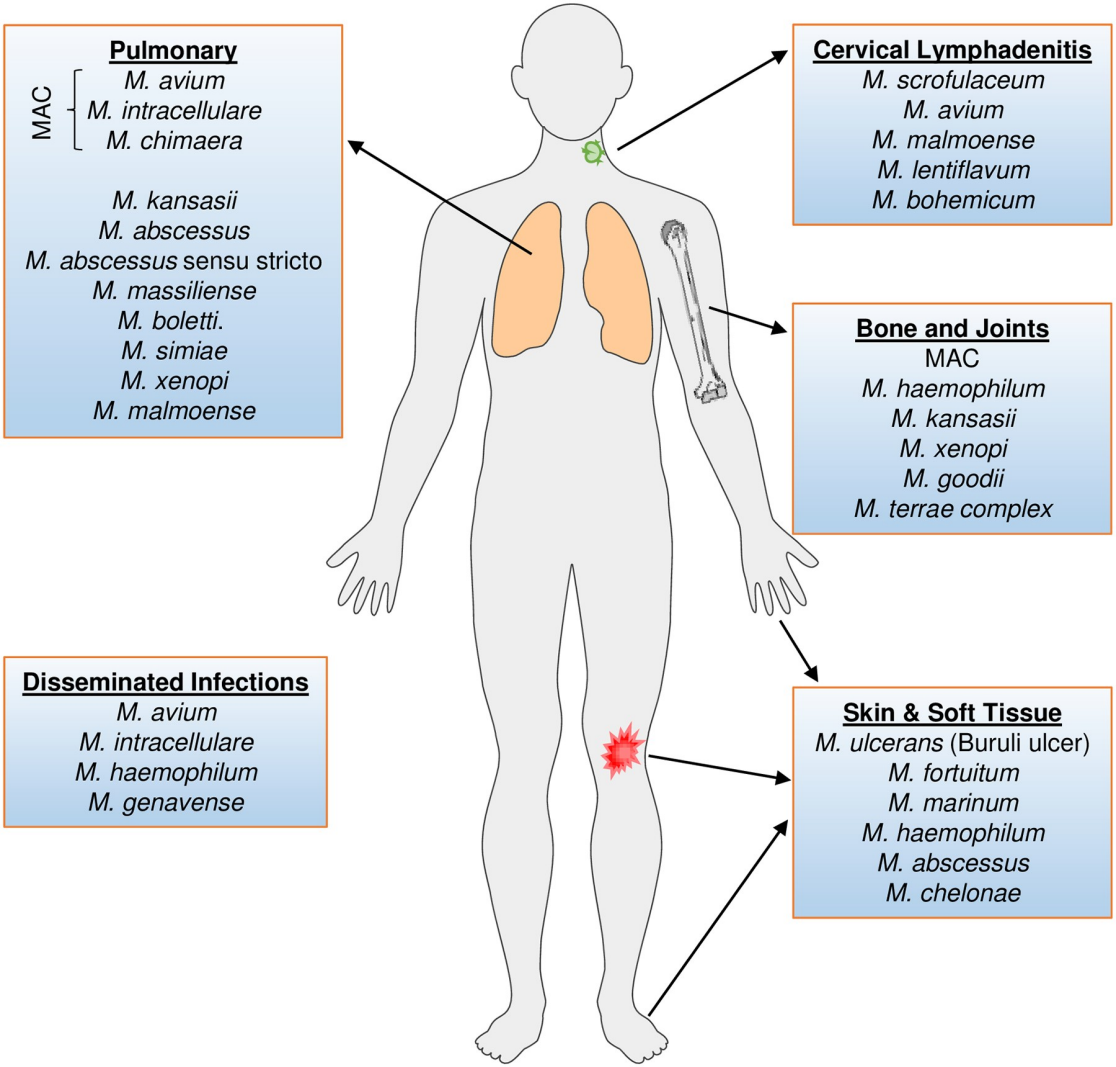
Body sites affected by NTM species. Pulmonary infections are generally due to inhalation from environmental sources. Disseminated infections are most prevalent in immunocompromised persons, such as those on anti-TNF antibody therapy or suffering from HIV. Cervical lymphadenitis presents most commonly in children. Bone and joint infections by NTM are usually introduced via trauma. Lastly, skin and soft tissue infections are initiated via surgery, trauma, or broken skin barriers contacting contaminated water. Figure represents more commonly encountered species; some less-common species are not depicted. HIV, human immunodeficiency virus; MAC, *M*. *avium* complex; NTM, nontuberculous mycobacteria; TNF, tumor necrosis factor.

The nontuberculous mycobacteria (NTM), defined as any mycobacterial pathogen other than *Mycobacterium tuberculosis* (*Mtb*) or *M*. *leprae*, include over 170 different species varying in their ability to cause disease [1]. While some species are implicated worldwide (for example, *M*. *avium* complex [MAC], *M*. *abscessus*), others (for example, *M*. *malmoense*) are regionally significant [2]. NTM are geographically heterogeneous and cause a spectrum of diseases that include tuberculosis (TB)-like pulmonary and extrapulmonary disease, cervical lymphadenitis in young children, and visceral and disseminated disease (Fig 1). Pulmonary NTM infections are most commonly due to MAC, *M*. *kansasii*, and *M*. *abscessus*, which cause a substantive, often unappreciated, worldwide burden of illness. Although NTM may cause disease similar to *Mtb*, they generally do not respond to classic TB drug regimens, and therefore a misdiagnosis of *Mtb* can lead to poor treatment, particularly in resource-poor settings lacking diagnostic infrastructure. NTM-associated disease is more abundant than previously believed and is a quietly unfolding disease epidemic, even overtaking TB prevalence in some areas [3, 4]. A recent nationwide study in a low-TB–incidence country was performed on over half a million mycobacterial cultures to determine the annual incidence of culture-verified NTM disease from 1991 to 2015 [5]. Higher than anticipated incidence rates of disease caused by NTM in the study by Hermansen and colleagues were observed in very young children 0–4 years of age (5.36/10^5^/year) and in older individuals (those aged 65–69; 2.39/10^5^/year) [5]. In the United States, an increased prevalence of NTM-associated lung disease cases in people above 65 years of age has also been observed [6]. This bimodal age association with NTM incidence eludes to the significant contribution of an insufficient immune response in susceptibility. Along with this overall increase in burden from NTM disease is the increase in direct medical costs associated with it, which are also staggeringly high. In 2010 alone, 815 million dollars were used to treat 86,244 cases of NTM in the United States [7]. Furthermore, NTM infection often leads to chronic disease that requires lengthy, complex, and sometimes poorly tolerated drug regimens over many months to years, and following treatment, patients can experience relapse from incomplete treatment or reinfection [8–13]. These studies and intricacies underscore the need to develop effective vaccines and drug treatments for use in highly susceptible populations and settings of emerging drug resistance [14].

Unfortunately, NTM infection and disease is not a reportable condition across much of the United States, and identification of NTM to the species level is not routinely done. Despite this variance in methodology and reporting across geographical areas, NTM prevalence has steadily risen since 1950 and is likely an underestimate [15]. The most common NTM species to cause lung disease belong to the MAC—composed primarily of *M*. *avium* and *M*. *intracellulare*. With high-throughput gene sequencing, several more related species have been identified that are under the MAC umbrella, including *M*. *chimaera* [16]. MAC species are most abundant across the Americas (85%–35.4%), Australia (83%–67.3%), Europe (82%–22.4%), and regions of Asia (71.4%–39.7%) compared to other species causing pulmonary disease [17]. Other frequently cultured NTM include *M*. *kansasii* and *M*. *abscessus*, whereas less frequent infections can occur with *M*. *xenopi*, *M*. *fortuitum*, and *M*. *chelonae* [18]. Whole-genome sequencing (WGS) of *M*. *abscessus* isolates is advancing our understanding of epidemiology, geographical diversity, and transmissibility [19, 20], and this process could be applied to other clinical NTM isolates. Despite an already large prevalence, species of the *Mycobacterium* genus are destined to increase further in the coming years; in fact, isolates not fitting any known species are frequently encountered in reference laboratories. This review will specifically highlight MAC and *M*. *abscessus* because they represent a significant proportion of disease worldwide. See the excellent reviews highlighting issues surrounding the diagnosis of NTM [21, 22], including this diverse representation of species in any given infection. Importantly, species-level identification of NTM has a large impact on treatment selection and success.

NTM organisms from environmental sources, including drinking and natural water, as well as soil and dust, can colonize human epithelia [23]. Furthermore, the steady increase of NTM infections is likely due to a wide contribution of factors, including an individual’s overall greater exposure to large-volume aerosols, a modernization of plumbing away from antibacterial copper pipes, and lower hot water temperatures, which may promote environmental colonization and NTM persistence. A comparative epidemiologic study evaluated water-aerosol–generating activities and found that these activities were not associated with MAC lung disease in human immunodeficiency virus (HIV)-negative adults; however, colonization of virulent isolates of MAC on faucets or associations in immunocompromised people, etc., were not ruled out by the authors as disease associations [24]. Indirect transmission is also an important epidemiological consideration, particularly for immunosuppressed individuals. Indeed, there is evidence that aerosolized NTM can also survive on fomites, providing another mechanism for spread, particularly for susceptible cystic fibrosis (CF) patients [25]. This nearly ubiquitous presence of NTM in the environment makes them ideal candidates for opportunistic infections and therefore warrants specific and detailed diagnostics and further evaluation for intervention against disease.

### Host–pathogen interactions

It is unclear whether the increase in NTM disease is in part due to a change in pathogen virulence over time. Development of preventive and treatment strategies against NTM will require a more comprehensive understanding of these dynamic host–pathogen interactions. While several host and pathogen factors are known to increase risk of NTM infection, greater knowledge pertaining to the molecular mechanisms underlying these factors will be required to develop more targeted therapy and also to develop assays that may be used in clinical and preclinical studies. Certain body morphotypes, gender, and the use of immunomodulatory drugs, such as steroids and tumor necrosis factor (TNF)-α blockers, are associated with a higher risk of NTM infection. Impaired ciliary function also predisposes individuals to NTM disease, and further research into the role of defects in airway clearance in mycobacterial invasion needs to be conducted [26]. The prescription and practice of mechanical airway clearance or airway clearance therapy has been observed to play an important role in reducing negative disease associations in bronchiectasis patients [27] but has not yet been evaluated for a larger role in preventing NTM reinfection. Specific genetic and treatment-induced host factors that increase risk of NTM infection are discussed below.

### Increased risk

Disease caused by NTM is influenced by a complex interplay between exposure and host-related factors. A matched case-control study demonstrated positive associations between MAC disease status and chronic obstructive pulmonary disease (COPD), severe pneumonia, steroids, and immunomodulatory drug use [24]. Other host factors contributing to NTM pulmonary disease (NTM-PD) include thoracic skeletal abnormalities and genetic disorders that predispose the patient to bronchiectasis and/or lung infections (for example, CF). While some host risk factors do overlap with those for *Mtb* infection, others starkly do not; for instance, TB is a relatively rare diagnosis in individuals with CF [28]. Similar to TB, a prominent risk factor for acquiring NTM disease is the immunocompromised status of an individual—due to either an inherited or acquired immunodeficiency—including those with HIV infection, cancer, organ transplants, and inflammatory diseases, such as rheumatoid arthritis treated with anti-TNFα therapy [26, 29, 30]. A recent case-control study revealed several compounding risk factors for NTM disease in rheumatoid arthritis patients in a TB-endemic area, including a history of TB, hypertension, diabetes, interstitial lung disease, COPD, and corticosteroid treatment [31]. Indeed, certain medications can also influence NTM infections, for example, inhaled corticosteroids causing immunosuppression increases the risk of acquiring NTM infections [24, 32, 33]. Although there is a greater incidence of both NTM and TB infections in immunocompromised HIV patients, there is an important dichotomy in the onset of mycobacterial disease with respect to HIV progression. There is great difficulty diagnosing TB in HIV-infected individuals; however, because of the high rates of extrapulmonary TB and frequency of smear-negative disease, it is evident that TB occurs relatively early in HIV infection [34]. Conversely, a recent US study observed NTM-disseminated disease in HIV patients, primarily due to MAC, with the highest incidence in those with cluster of differentiation 4 (CD4^+^) T-cell counts less than 50 cells/mm^3^ [35].

Clustering of disease within families suggests a heritable genetic predisposition to disease susceptibility [17, 36–38]. These heritable factors largely align with host immune responses to infection, which are also well documented for *Mtb* infections. For example, interferon-γ (IFN-γ) is an important host response against NTM in both mice and humans; disseminated NTM disease occurs in mice deficient in either IFN-γ receptor (IFNγR) or IFN-γ [39]. Additional immune deficiencies such as polymorphisms in interleukin 12 (IL-12) (IL-12p40 [40], IL-12 receptorβ1 [41], IFN-γ receptor [IFNγR1] [42] and IFNγR2 [43]) genes and natural-resistance–associated macrophage protein 1 gene (*NRAMP1*) are risk factors for NTM infection [26]. Interestingly, levels of anti-IFN-γ and anti-granulocyte–macrophage colony-stimulating factor (GM-CSF) autoantibodies are higher in patients with NTM-PD [44]. As previously discussed, host age is also an important and significant factor for acquiring NTM disease. A retrospective cohort study of NTM disease in Denmark showed a relative risk of a positive NTM culture in the elderly (>65 years) greater than 10 times that of adolescents (aged 15–19 years) [5]. Pulmonary NTM infection in the US occurs mostly in tall, thin, nonsmoking postmenopausal white women who have a complex condition involving additive genetic variants in immune, ciliary, connective tissue, and CF transmembrane conductance regulator (CFTR) genes [38]. The disease in this population, sometimes referred to as “Lady Windermere syndrome,” presents with nodular bronchiectasis frequently involving the right middle lobe or left lingula and tends to have a much slower progression than cavitary disease, such that long-term follow-up (months to years) may be necessary [45, 46]. Affected host immune status, genetic polymorphisms, and age thus all play prominent roles in NTM susceptibility.

Beyond host factors, the distinct phenotypes and variability between NTM species can also influence disease states. The ability of NTM to form different morphotypes and robust biofilms [47–52] may play a role in pathogenesis by enabling bronchial epithelial attachment and respiratory infection [53], whereas *Mtb* biofilms may be involved in the formation of caseous necrosis and cavitation within the lung [47, 49, 50]. Specifically for *M*. *abscessus*, the in vitro zebrafish model has revealed smooth (expressing glycopepidolipid [GPL]) and rough (loss of GPL) phenotypes have divergent effects on infected macrophages that induce either protective granulomas and chronic infection or abscess formation and acute infection, respectively [48]. The recognition and characterization of different NTM morphotypes were established in the 1950s, but the mechanism(s) underlying colony phenotype and relationships to pathogenicity is incompletely understood for many NTM species [53, 54].

### Other NTM species of clinical importance

Other clinically important species of NTM include, but are not limited to, *M*. *kansasii*, *M*. *haemophilum*, *M*. *marinum*, and *M*. *ulcerans* (Fig 1). Some of these NTM infect the skin, often occurring following procedures such as tattooing, liposuction, surgical procedures, or trauma, all of which introduce the mycobacteria into the disrupted skin barrier. *M*. *ulcerans* infection (Buruli ulcer) is prevalent in sub-Saharan Africa and some parts of Australia and is usually introduced by trauma from contaminated surfaces and water. Increased risk of mycobacterial infections (including NTM infection) can be attributed to antirheumatic medications, such as anti-TNF therapy [55]. Specifically, monoclonal antibody therapy with TNF inhibitors used for the treatment of psoriasis can be a risk factor for skin infections with *M*. *fortuitum* [56]. Anti-TNF therapy can also enhance risk of infection with *M*. *intracellulare* and *M*. *avium* pathogens [57]. *M*. *kansasii* causes lung disease that is clinically indistinguishable from TB and is inherently resistant to standard first-line drugs, such as pyrazinamide [58], used against *Mtb*. Many of the 170 and counting NTM species have variable but relevant clinical impact and should be identified for successful treatment.

### Clinical diagnosis of adult pulmonary NTM infections

Humans encounter environmental mycobacteria with variable clinical disease relevance on a daily basis. Therefore, a single positive culture from nonsterile sources including the respiratory or digestive tract does not necessarily indicate infection or disease and makes treatment decisions less straightforward. Further complicating diagnosis are the nonspecific symptoms of NTM-PD, including a chronic cough, with or without sputum production or hemoptysis, and progressive fatigue or malaise. Weight loss, fever, and night sweats are less frequent—occurring in 30% to 50% of patients—and often indicate advanced disease [59]. Clinical diagnosis of NTM-PD involves the presentation of cough, fatigue, and often weight-loss symptoms, accompanied by positive radiographs and isolation of an NTM from clinical specimens such as sputum [22]. Often, 2 positive microbiological cultures are needed to differentiate NTM disease versus colonization [21, 60]; however, these criteria have not been validated with respect to progression to disease [61, 62]. The exclusion of other infectious (for example, TB, nocardiosis, and fungal infection) and noninfectious diseases (for example, sarcoidosis) is also critical when diagnosing NTM. Clinical, radiographic, and microbiologic criteria are equally important, and all must be met to make a diagnosis of NTM disease.

### Microbiology and molecular identification of NTM

Early control of mycobacterial infections is dependent on methods for rapid identification of complex NTM and *Mtb* infections from a clinical sample and can reduce both morbidity and mortality through implementing the best course of drug treatment. The method for detection of NTM typically involves smear microscopy and culturing specimens, such as sputum, on solid and/or liquid media [63], while the preferred staining procedure is fluorochrome microscopy [64]. To the extent that it is possible, NTM should be identified to the species level using tools such as commonly employed line-probe assays, which can also be used to amplify drug-resistance–determining regions [63]. The GenoType NTM-DR line-probe assay (Hain Lifescience, Nehren, Germany) is one such tool recently described for the identification of clinical *M*. *abscessus* subspecies (subsp.) and drug resistance [65]. Rapid species identification can also be determined using commercial DNA probes (MAC, *M*. *kansasii*, and *M*. *gordonae*), while group- or complex-level identification can be accomplished with high-performance liquid chromatography (HPLC). For some NTM isolates, especially rapidly growing mycobacteria (RGM; *M*. *fortuitum*, *M abscessus*, and *M*. *chelonae*), extended antibiotic in vitro susceptibility testing, DNA sequencing, or polymerase chain reaction (PCR) restriction endonuclease assay (PRA) may be necessary. Another assay available for the detection of *Mycobacterium* species from clinical samples is a PCR-reverse blot hybridization assay (REBA) Myco-ID assay (YD Diagnostics, Yongin, South Korea), in which multiple targeted oligonucleotide specific probes (*Mycobacterium*-species specific) are bound to a nitrocellulose membrane strip, then hybridized with biotinylated PCR products and subsequently visualized by colorimetric hybridization signals [66, 67].

Molecular identification of different NTM species can also be accomplished via genomic DNA comparison, though this process is labor intensive. A dramatic shift and update in mycobacterial taxonomy came with the ease of DNA sequencing. Early investigations demonstrated that the mycobacterial 16S rRNA gene is highly conserved [68], that it is more accurate than phenotypic methods for species identification [69], and that differences in the sequence of 1% or greater can generally define a new species [70]. Furthermore, in a large study, molecular-based PCR methods were 84.7% sensitive at detecting NTM species from clinical samples, while phenotypic-culture–based methods only reached 78.0% and required significantly more time to complete [71]. For these reasons, 16S rRNA can be used as a standard reference when comparing detection techniques. Recently, Rodríguez-Sánchez and colleagues assessed 125 NTM isolates using matrix-assisted laser desorption ionization-time of flight (MALDI-TOF) mass spectrometry, the GenoType common mycobacteria (CM)/additional species (AS) assay, and a 16S rRNA/*hsp*65 gene sequencing reference assay to determine the alignment of these different techniques [72]. The MALDI-TOF assay was in agreement with the reference assay in 118/125 cases (94.4%), and the GenoType CM/AS assay was in agreement in 105/125 cases (84%), showing some limitations in loss of sensitivity of the GenoType CM/AS assay [72]. Conversely, while the MALDI-TOF assay requires a mass spectrometer, the GenoType CM/AS assay can be performed using either manual or automated processing, making it more accessible in resource-limited settings. Besides the aforementioned assays, another innovative molecular assay, the Quantamatrix multiplexed assay platform (QMAP) system recently described by Wang and colleagues [73], allows clinicians to discriminate between mycobacterial species. This assay utilizes an automated magnetic-bead–based assay following similar PCR steps as used in the PCR-REBA assay, except denatured biotinylated PCR products are added to species-specific oligonucleotide probes coupled to carboxylated microdisks, followed by the addition of streptavidin R-phycoerythrin conjugate and automated reading of fluorescence intensity. The specificity of the QMAP system was assessed from 295 mycobacterial respiratory clinical isolates, several *Mycobacterium* reference strains (including *Mtb* H37Rv and NTM strains), and non-*Mycobacterium* strains. This process yielded high specificity and sensitivity in a short period (3 hours). The overall percentage agreement between the QMAP system and PRA Myco-ID and REBA Myco-ID was 92.8% and 100%, respectively. Molecular typing methods *of Mtb* and NTM species have been thoroughly described in a recent review [74], including assay expense, requirement of specialized equipment and trained staff, reproducibility, specificity and sensitivity, and the time required for an accurate diagnosis. Correct identification of NTM species is dependent on an extensive and routinely updated molecular database and is critical for successful treatment of NTM infection.

### Pulmonary NTM infection and treatment

The treatment and outcomes of NTM diseases differ depending on the NTM species, and therefore species-level NTM identification is clinically important. Standard treatment of infection with slow-growing NTM (such as MAC-PD) in immunocompetent patients involves the combination of macrolides (azithromycin [AZM], chemically considered an azalide, or clarithromycin [CLR]), ethambutol (EMB), and rifampin (RIF) [21]. Macrolides act by binding to the peptide exit tunnel of the ribosome, thus preventing the peptide chain from exiting the peptidyl transferase center of the ribosome. MAC species are notorious for developing drug resistance to many antibiotics through modifications of the drug-binding site to reduce binding of the agents, as described in a recent review [75], making infections exceedingly difficult to treat. The most important risk factors for developing macrolide-resistant MAC are macrolide monotherapy and the combination of macrolide and fluoroquinolone without a third companion drug. For patients with fibrocavitary MAC lung disease or severe nodular/bronchiectatic disease, regimens that include both macrolides, streptomycin, and aminoglycosides such as kanamycin and amikacin that bind to the bacterial 30S ribosomal subunit and induce cell death are recommended [21].

The American Thoracic Society provides drug guidelines and recommended regimens for NTM, including those based on the severity and disease status of NTM infections [21]. The British Thoracic Society has also recently published guidelines for the management of NTM-PD [60]. A recent review outlines dosages and common adverse events attributed to several drugs used for treatment of NTM-PD [76]. There are a number of toxicities and side effects noted for drugs typically used to treat MAC that could affect treatment compliance, such as CLR-induced headache, nausea, and vomiting or rifamycin-induced anemia, hepatotoxicity, lymphocytopenia, rash, and/or thrombocytopenia [76]. The genetic predisposition to antimicrobial resistance as well as the poor tolerability of the current standard regimens makes the discovery and development of novel drugs for NTM-PD, including both *M*. *abscessus* and MAC infections, a research priority. Furthermore, classifying and standardizing treatment success and outcome reporting for comparative assessments may help better identify positive versus negative treatment regimens [14].

Clinicians also have to consider whether a positive culture in the absence of overt symptoms may suggest colonization versus infection, and the significant extent of side effects from prolonged treatment is important when evaluating the need to treat. Indeed, over half (63%) of patients with MAC-PD in a recent study progressed, and these cases correlated with a higher bacterial load and pulmonary destruction. The remaining 47% of MAC-PD patients remained stable [77], and in some cases, those who are untreated revert back to negative cultures spontaneously. Treatment decisions rely on evaluating whether the patient is symptomatic, whether they present with background genetic or pharmacologic factors that would enhance susceptibility, and the extent of radiographic abnormalities, which suggest evidence of disease progression. Further assessment of specific biomarkers that correlate with progression to disease is critical and an active area of research.

### Pulmonary MAC and *M*. *abscessus* subsp. in the CF patient

The rapidly growing *M*. *abscessus* subsp. (including *M*. *abscessus* subsp. *abscessus*, *M*. *abscessus* subsp. *massiliense*, and *M*. *abscessus* subsp. *bolletii*) are emerging pathogens, with outbreaks in surgical patients in Brazil and in CF patients in the United Kingdom [25, 78, 79]. The prevalence of NTM in CF patients, a major health threat, has been steadily increasing since the early 1980s and is estimated to be 7%–13% in this population [28]. Recommendations to enable better NTM-centric healthcare for CF patients have been put forth by the CF Society and the CF Foundation [80]. These recommendations, although not exhaustively listed here, include using sputum but not oropharyngeal swabs for NTM screening, using both solid and liquid cultures for a minimum of 6 weeks for respiratory samples, using molecular identification of NTM isolates, testing susceptibility of MAC to CLR prior to treatment, testing sputum samples for NTM culture every 4–8 weeks throughout the course of treatment, and prescribing NTM antibiotic therapy for 12 months beyond culture conversion [80]. Additional treatment strategies that are relevant for CF patients should be investigated, such as identifying immune pathways that may be leveraged to help those with this disease.

### Treatment for *M*. *abscessus* subsp. infection

RGM infections can present as an asymptomatic, inert disease with minimal clinical symptoms to severe bronchiectasis and cavitary pulmonary disease with substantial morbidity and mortality. The many subsp. of *M*. *abscessus* [81] are a classic example of how species and subsp. identification in an NTM infection can dramatically affect treatment selection and outcomes [12]. Molecular classification can help inform researchers and clinicians about variations in transmissibility, pathogenesis, and drug sensitivity, all within a single species such as *M*. *abscessus*. Generally, *M*. *abscessus* subsp. *abscessus* infections are more severe and difficult to treat. CLR and AZM are the standard therapeutic agents for *M*. *abscessus* subsp. *massiliense*, which lacks a functional, active, inducible-macrolide–resistance gene, erythromycin ribosomal methylase (*erm)* gene [82]. The *erm* gene—dominant in *M*. *abscessus* subsp. *abscessus*, *M*. *abscessus* subsp. *bollotii*, and *M*. *fortuitum* strains (but not in *M*. *chelonae*)—promotes methylation of the 23S rRNA, rendering the bacteria resistant against macrolides, lincosamides, and streptogramins. Induction of this gene is not detected by conventional susceptibility testing, however, since this requires extended culture incubation observation for up to 2 weeks. Once samples are cultured for susceptibility over 2 weeks, species and subsp. can be identified as well as helping dictate decisions about the makeup and length of treatment [60]. Mutational resistance is also observed in the ribosomal L4 protein (*rrl*) encoding gene of some *M*. *abscessus* subsp. The high propensity for macrolide resistance underscores why the use of monotherapy for infections caused by RGM is not recommended [21]. There is active research to develop rapid diagnostic tests for these inducible and mutational-based mechanisms of drug resistance [83], which is severely needed to replace lengthy, extended-culture–based methods. Furthermore, species and subsp. identification of NTM can help predict whether there is a higher propensity for recurrence, relapse, or reinfections [8, 84–86], which will also play a part in determining treatment courses.

### Experimental animal models for NTM

As discussed above, there are diverse factors that affect NTM disease, including host factors and innate mycobacteria phenotypes and antibiotic resistance. Preclinical animal models can be leveraged to study the influence of these factors and evaluate novel therapeutic drugs and regimens for treatment of NTM infections. Two major categories of NTM disease to consider for animal model development include pulmonary disease and extrapulmonary-disseminated disease (typically presenting in those who are immunocompromised). NTM are generally less virulent than *Mtb*, and therefore the capacity to induce a sustained progressive infection in a mouse strain is an important criterion and current hurdle for the development of an experimental mouse model. Previous studies have shown that most immunocompetent mouse strains serve as outstanding models for the more virulent MAC species but demonstrate rapid clearance when infected with the less virulent *M*. *abscessus* isolate [87], making model development and selection challenging (Tables 1 and 2). Many different mouse strains have been used to screen different drug compounds against MAC, including CLR, RIF, rifapentine (RPT), moxifloxacin (MXF), EMB, and amikacin (AMK) (Table 1). Other diverse preclinical models are available for testing *M*. *abscessus* pathogenesis and in vivo activity of drug compounds against *M*. *abscessus*, including amoebae, *Drosophila melanogaster*, and zebrafish embryo models [48]. Exploiting the host-specific conditions required for the successful growth and pathogenicity outcome of each NTM organism will enable the testing and development of treatment strategies in an appropriate preclinical model.

**Table 1:** Mouse strains/models used for MAC infection.

Mouse strain	*M*. *avium* route of infection and dose	Productive infection (organ)	Compound screening	Reference
C57BL/6	aerosolization; approximately 10^5^/mouse	lung and spleen	CLR	[100]
C57BL/6 (modified Cornell-like model)	*M*. *avium* complex strains; aerosolization (500–10^4^ CFU/ mouse)	lung	CLR-RIF	[103]
C57BL/6	*M*. *avium* subsp. *hominissuis*; intranasal 2.25 × 10^7^ CFU/mouse	lung	LAI	[150]
C57BL/10	intranasal; 10^5^ CFU/mouse	lung, spleen, and liver	N/A	[101]
129Sv	IV; 10^6^ CFU/mouse	lung, spleen, and liver	N/A	[96]
BALB/c	aerosolization; approximately 10^5^ CFU/mouse	lung and spleen	CLR, CLR-EMB-RIF, MXF-EMB-RIF, CLR-MXF-EMB-RIF	[100]
iNOS^-/-^	IV; 10^6^ CFU/mouse	liver	N/A	[151]
iNOS^-/-^	IV; 10^6^ CFU/mouse	lung, spleen, and liver	N/A	[96]
TNFα p55 receptor^-/-^	IV; 10^6^ CFU/mouse	lung and spleen	N/A	[152]
Beige	aerosolization; approximately 10^5^ CFU/mouse	lung and spleen	CLR	[100]
Beige	IV; 10^6^ CFU/mouse	lung, spleen, liver, and gut	N/A	[90]
Nude	aerosolization; approximately 10^5^ CFU/mouse	lung and spleen	CLR, CLR-EMB-RIF, MXF-EMB-RIF, CLR-MXF-EMB-RIF	[100]

**Abbreviations**: CFU, colony-forming units; CLR, clarithromycin; EMB, ethambutol; iNOS, inducible NO synthase; IV, intravenous; LAI, liposomal amikacin for inhalation; MXF, moxifloxacin; N/A, not applicable; NO, nitric oxide; RIF, rifampin; TNF, tumor necrosis factor.

**Table 2:** Mouse strains used for *M*. *abscessus* infections and drug screening.

Mouse strain	NTM used and route of infection	Productive infection (organ)	Compound screening	Reference
C57BL/6	*M*. *abscessus*; aerosolization (HDA and LDA)	HDA (lungs and spleens), LDA (no)	N/A	[94]
C57BL/6	*M*. *abscessus* and *M*. *massiliense*; IV (4 × 10^6^ to 10^7^)	lung	CLR, MXF, CLR/MXF, AZM, AZM/MXF	[153]
BALB/c	*M*. *abscessus*-R; IV (10^4^/mouse)	lungs and spleen	N/A	[87]
Ob/Ob	*M*. *abscessus*; aerosolization (HDA and LDA)	HDA (lungs and spleen), LDA (no)	N/A	[94]
CF mouse	*M*. *abscessus*-R and -S morphotype; IT (1.6 × 10^6^/mouse)	yes	N/A	[154]
iNOS^-/-^	*M*. *abscessus*; IV (10^6^/mouse)	lungs, spleen, and liver	N/A	[87]
Cybb^-/-^	*M*. *abscessus*; IV (10^6^/mouse)	lungs, spleen, and liver	N/A	[87]
TNFα receptor^-/-^	*M*. *abscessus*; IV (10^6^/mouse)	lungs, spleen, and Liver	N/A	[87]
MyD88^-/-^	*M*. *abscessus*; IV (10^6^/mouse)	lungs, spleen, and liver	N/A	[87]
C3HeB/FeJ	*M*. *abscessus*; IV (10^6^/mouse)	lungs, spleen, and liver	N/A	[87]
Beige	*M*. *abscessus*; IV (10^6^/mouse)	lungs, spleen, and liver	N/A	[87]
GKO	M. *abscessus*; aerosolization	LDA or HDA lungs and spleen	N/A	[94]
GKO	*M*. *abscessus*; IV (10^6^/mouse)	lungs, spleen, and Liver	CLR, CLF, BDQ, CLF/BDQ, CIP, AMK	[87]
GKO	*M*. *abscessus* subsp. *massiliense*	lungs and spleen	N/A	[155]
GM-CSF^-/-^	*M*. *abscessus*; aerosolization (10^6^/mouse)	lungs and spleen	AZM	[156]
GM-CSF^-/-^	*M*. *abscessus*; IV (10^6^/mouse)	lungs, spleen, and liver	N/A	[87]
Nude	*M*. *abscessus*; IV (10^6^–10^8^/mouse)	lungs, spleen, liver, and kidneys	FOX, AMK, CLR, FOX/AMK/CLR, TGC	[157]
Nude	*M*. *abscessus*; IV (10^6^/mouse)	lungs, spleen, and liver	N/A	[87]
SCID	*M*. *abscessus*-R; IT (10^4^/mouse)	lungs and spleen	N/A	[158]
SCID	*M*. *abscessus*; IV (10^6^/mouse)	lungs, spleen, and liver	CLR, BDQ, CLF, BDQ/CLF	[87]

HDA = approximately 1,000 bacilli/mouse, and LDA = approximately 100 bacilli/mouse. **Abbreviations**: AMK, amikacin; AZM, azithromycin; BDQ, bedaquiline; CF, cystic fibrosis; CIP, ciprofloxacin; CLF, clofazimine; CLR, clarithromycin; FOX, cefoxitin; GKO, IFN-γ knockout; GM-CSF, granulocyte-macrophage colony-stimulating factor; HDA, high-dose aerosol; IFN-γ, interferon-γ; iNOS, inducible NO synthase; IT, intratracheal; IV, intravenous; LDA, low-dose aerosol; MXF, moxifloxacin; N/A, not applicable; NTM, nontuberculous mycobacteria; R, rough morphotype of *M*. *abscessus*; S, smooth morphotype of *M*. *abscessus*; SCID, severe combined immunodeficiency; TGC, tigecycline; TNF, tumor necrosis factor.

### Animal models for slow-growing mycobacteria

The Beige mouse is used as a standard model for MAC disease [88]. This mouse model was developed in the mid-1990s because of the increasing numbers of HIV-seropositive patients becoming coinfected with *M*. *avium* [89]. Beige mice display many immune deficiencies similar to those occurring in AIDS patients [88], as well as a susceptibility to infection with NTM following either intravenous or aerosol infection, providing a unique opportunity for the study of MAC infections in this model [88–92] (Table 1). Additionally, Beige mice exhibit a deficiency of neutrophil influx at the site of infection that increases their susceptibility to *M*. *avium*, which is rescued by the adoptive transfer of neutrophils from wild-type mice [90]. Corresponding neutrophil-depletion studies with C57BL/6 mice show increased susceptibility to *M*. *avium* [90]. The initial discovery and further confirmation of MAC disease in the Beige mouse model have encouraged many investigators to use this model to screen the chemotherapeutic potential of promising compounds for the treatment of MAC disease. Importantly, many chemotherapeutic compounds against MAC disease and other NTM that have shown activity in the Beige mouse model have been successfully translated into clinical application [93, 94]. Therefore, the Beige mouse is an excellent immunocompromised model for NTM infections and compound screening.

Preclinical models can also be used to study mechanisms of host immunity against mycobacterial infections. For example, the effects of nitric oxide (NO) on *Mtb* and *M*. *avium* infection in mice have been well studied. Through these investigations, it has been demonstrated that NO contributes to protective host responses against *Mtb* in the murine model, both in vitro (in macrophages) and in vivo [95]. However, interesting differences are present in terms of NO and NTM infections. Studies using mice devoid of inducible NO synthase (iNOS^-/-^) show improved control of *M*. *avium*, denoted by reduced mycobacterial burden in multiple organs compared to wild-type 129Sv mice [96]. Furthermore, treatment of bone-marrow–derived macrophages with IFN-γ and TNFα from either wild-type or iNOS^-/-^ mice leads to reduced intracellular *M*. *avium* burden. These results suggest that the protective effects of IFN-γ and TNFα against NTM are not mediated by the production of NO. NO has also been suggested to have a regulatory role on IFN-γ–expressing T helper 1 (T_H_1) CD4^+^ T cells because iNOS^-/-^ mice show greater IFN-γ production and increased CD4^+^ T-cell numbers [97]. The mechanism by which NO is protective against *Mtb* and not against NTM in mouse models of infection remains poorly understood [98]. One study using an intratracheal infection with *M*. *avium* showed similar kinetics of cytokine gene expression between TNFα and iNOS, in which expression levels in the lung were low early in infection when bacterial load was higher and cytokine expression was higher late in infection when bacterial levels were lower, suggesting a possible role of NO against *M*. *avium* [99].

Evaluating multiple strains of mice can also provide valuable insight into host immune responses and NTM pathogenesis. For example, several mouse strains were compared to evaluate responses following an *M*. *avium* aerosol challenge, including 2 immunocompetent mouse strains (BALB/c, and C57BL/6) and 2 immunodeficient mouse strains (Nude and Beige mice). Not surprisingly, Nude mice showed the greatest susceptibility to *M*. *avium* infection compared to the other mouse strains, whereas drug treatment efficacy was determined to be most successful in BALB/c mice [100]. In the same study, C57BL/6 mice showed the greatest resistance to *M*. *avium* 8 weeks postchallenge. C57BL/10 mice depleted of CD4^+^ T cells and infected using an intranasal infection of *M*. *avium* clearly support a role for CD4^+^ T cells [101]. Furthermore, IFN-γ depletion before and during *M*. *avium* infection leads to increased bacterial burden in the lung, spleen, and liver, suggesting a protective role for IFN-γ against this pathogen [101].

Like *Mtb* [102], NTM exposure can result in acute and chronic infections, as well as reactivation post-treatment [8]. Preclinical modeling of these different disease states may help isolate treatment options, including harnessing host immunity. NTM infection reactivation has been studied using a modified Cornell-like murine model (typically used to study reactivation of latent *Mtb*) and has successfully been developed for experimental reactivation of pulmonary MAC infection (Table 1), [103]. In this model, C57BL/6 mice are infected by the aerosol route with MAC strains and are treated with CLR and rifampicin for 6 weeks, and then treatment is stopped. Twelve weeks following drug treatment, mice are given immunosuppressants (dexamethasone or sulfasalazine) for 5 weeks to expose any remaining bacteria post-treatment. Bacterial burden is assessed in the organs at different times after immunosuppression to measure reactivation. This model could also be useful for determining the potential efficacy of combined drug and immunotherapy regimens by quantifying the numbers of bacilli remaining after treatment.

### Animal models for RGM

The most clinically important RGM to cause human lung disease belongs to *M*. *abscessus*. *M*. *abscessus* subsp. *abscessus* and *M*. *abscessus* subsp. *bollettii* have a functional *erm41* gene; therefore, resistance to macrolides may be identified. It has been challenging to develop an animal model for screening compounds against RGMs because of gaps in fully understanding their pathogenesis of infection and relative avirulence. One study [94] has shown, for example, that C57BL/6 and leptin-deficient (Ob/Ob) mice challenged with a low-dose aerosol (LDA, approximately 100 bacilli per mouse) of *M*. *abscessus* did not develop a progressive infection. Additional studies using a high-dose aerosol (HDA, approximately 1,000 bacilli per mouse) in C57BL/6 and Ob/Ob mice have resulted in an established infection and an early pulmonary influx of IFN-γ^+^ CD4^+^ T cells. This early influx of CD4^+^ T cells producing IFN-γ resulted in clearance of *M*. *abscessus* in both mouse strains. It is important to note that mycobacterial elimination was delayed in the Ob/Ob mice, demonstrating an increase in susceptibility to infection in this mouse model. Conversely, IFN-γ knockout (GKO) mice challenged with a LDA or HDA of *M*. *abscessus* showed progressive lung infection, associated with the influx of T cells, macrophages, and dendritic cells and subsequent granuloma development, despite a lower bacterial load [94]. In addition, HDA challenge of the GKO mice induced IL-4 and IL-10, producing CD4^+^ and CD8^+^ T cells within the lungs, capable of suppressing of protective immunity. The thorough and systematic evaluation of host immune factors influencing NTM infection progression and disease can be achieved with selective mouse modeling and comparisons across strains.

A progressive aerosol infection model has proven elusive since most mouse models with significant deficits in innate or acquired immunity are still able to clear an infection with a high level of RGM. This highlights the need for improved understanding of the NTM pathogenesis of infection [87]. Importantly, even some mouse strains with specific defects in innate or acquired immunity infected with 1 × 10^6^ *M*. *abscessus* intravenously were able to control the infection [87]. Mouse strains capable of bacterial clearance include Beige (dominant T_H_2 immunity), iNOS^-/-^, Cybb^-/-^ (devoid of superoxide generating enzyme), TNFα receptor (R)^-/-^, C3HeB/FeJ, GKO, and MyD88^-/-^ mice. During chronic infection (40 days), *M*. *abscessus* was still present at levels below the initial inoculum in the lungs of the C3HeB/FeJ, GKO, and MyD88^-/-^ mice. Moreover, the GKO and MyD88^-/-^ mice maintained viable but reduced levels of *M*. *abscessus* in the spleen and liver after 40 days. SCID, Nude, and GM-CSF^-/-^ mice infected intravenously with *M*. *abscessus* demonstrated sustained or progressive bacterial burden [87], which supports an important role for T cells and GM-CSF–dependent cell phenotypes in protective immunity against *M*. *abscessus* and other rapid growing subsp.

Acute and chronic compound efficacy have been tested in the *M*. *abscessus*-infected SCID mice (Table 2), [87]. Single and combination treatment studies using standard anti-NTM compounds (CLR, clofazimine [CLF], AMK, ciprofloxacin [CIP], bedaquiline [BDQ], and CLF-BDQ) have been completed. An advantage of using severely immunocompromised mice (SCID, Nude, and GM-CSF^-/-^) for modeling *M*. *abscessus* infection is the presence of foamy cells in the lungs after 40 days of infection, a cellular phenotype commonly seen in the histopathologic specimens of human NTM lung disease [87]. Murine models of NTM infection are capable of developing non-necrotic and necrotizing granulomas, mimicking diverse pulmonary pathology in NTM patients. Additional models such as the embryonic zebrafish have been developed to assay *M*. *abscessus* [104] for rapid compound screening, while a hollow-fiber model has been utilized for RGM compound screening [87]. The biggest challenge that remains to advance our knowledge in NTM pathogenesis and protection is to fully understand the process of human NTM infections (environmental, nosocomial, and endogenous/exogenous reinfection), which would allow us to better emulate these processes in our animal models.

### Vaccines for NTM

Despite the significant global impact of opportunistic NTM and the need to stop subsequent reinfection from the environment in susceptible hosts [105, 106], there are no vaccines currently available against these pathogens. However, various approaches may be exploited to develop and advance vaccines against NTM [107–110]. For example, similar virulence factors are expressed during infection with other pathogens, and cross-reactive antigens may afford induction of protective immunity against NTM [108]. This important gap in healthcare can be closed with the appropriate tools (models) and experience to develop a candidate vaccine targeting mycobacterial infections [111]. A therapeutic vaccine could also help overcome issues with acquired drug resistance to drugs such as CLR, following treatment against both slow-growing mycobacteria (SGM) including MAC [112] and RGM such as *M*. *abscessus* [113, 114]. For immunotherapeutic vaccine development, the NTM field can borrow strategies currently being used and evaluated for efficacy against *Mtb*. For example, our group has engineered a vaccine against *Mtb*, ID93 (a clinical TB antigen) + glucopyranosyl lipid adjuvant (GLA) formulated in an oil-in-water stable nanoemulsion (SE), which is currently being tested in a Phase 2a trial in South Africa for safety, immunogenicity, and dose selection in 60 TB patients administered post-treatment [115]. Escalating preclinical studies with ID93 + GLA-SE have demonstrated protective and therapeutic efficacy [116–122], and clinical results suggest the vaccine is safe and immunogenic [120, 122]. This strategy hinges on optimizing mycobacterial antigen selection and an immune-stimulating adjuvant, the synthetic Toll-like receptor 4 (TLR4) GLA-SE, capable of enhancing adaptive immune responses to many infectious pathogens [123]. Numerous preclinical and clinical vaccine studies for infectious diseases, allergy, and cancer [117, 124–141] have used a protein/adjuvant strategy that could be emulated for the development of vaccines targeted against NTM infections. Although correlates of protection have been elusive for mycobacterial diseases, more and more recent success has emerged from the TB vaccine community. This includes a cutting-edge correlate of risk RNA blood signature that predicts treatment outcomes and relapse in certain populations [142], providing a new benchmark for other vaccine candidates to reach, as well as recently published data demonstrating a protein antigen with immune-stimulating adjuvant (M72/AS01E) candidate vaccine produced 54% efficacy of preventing active TB in adults in a Phase 2b clinical trial [143]. As the TB research community continues to make strides in developing and testing vaccine candidates, these successes and tools should and could be cross-applied to the NTM field.

Variable host factors and mycobacterial experience are particularly important considerations for rational anti-NTM vaccine design. It has long been hypothesized that NTM-based preexisting immunity could lead to variability and/or lack of Bacillus Calmette–Guérin (BCG) vaccine efficacy [144]. Conversely, if BCG is given prior to NTM exposure in a mouse model, there is some protection against *M*. *avium* and *M*. *kansasii* [145]. BCG was ineffective against *M*. *intracellulare* and *M*. *simiae*, perhaps because BCG lacks cross-reactive antigens against these NTM [145]. Fraga and colleagues demonstrated transient protection (delayed onset of bacterial growth and foot swelling) against *M*. *ulcerans*, including increased CD4^+^ T_H_1 responses, following vaccination with BCG [146]. Recently, unexpected cross-reactivity of NTM/*Mtb*-specific chemokine (C-X-C motif) (CXCR3^+^ and CCR6^+^, respectively) memory T cells in non-TB–exposed healthy control (HC) donors has been observed [147]. The possibility of “boosting” NTM T-cell responses in NTM-exposed individuals with protein/adjuvant vaccines, either alone or as an adjunct to drug treatment, is currently being explored by our team. Adjuvant is likely to play a role in a robust vaccine response against several NTM, including the well-characterized T_H_1-inducing adjuvant GLA-SE [123]. For example, early IFN-γ induction has been shown to be critical for immunity against *M*. *ulcerans* infection, as shown by delayed progression of infection and reduced bacterial burden in wild-type compared to GKO mice [148]. Other data demonstrate promising protective responses in mice, including increased survival, decreased pathology, and reduced bacterial load compared to BCG, using a recombinant BCG-expressing *M*. *ulcerans* Ag85A in the Buruli ulcer mouse model [149]. Progress towards an effective NTM vaccine strategy will require careful consideration of the influence(s) of historical NTM exposure in addition to BCG or *Mtb* experience for possible immune interference or boosting effects and should be a major priority for the research community.

## Conclusion

Challenges facing the prevention and treatment of NTM disease include inadequate research—in part no doubt due to insufficient funding—the lack of knowledge of factors leading to susceptibility in mouse strains with different genetic backgrounds, and a poor understanding of the mechanisms utilized by NTM that allow for the evasion of host immunity resulting in bacterial persistence. By addressing these challenges, more effective prophylactic and therapeutic approaches for the prevention and treatment of NTM will likely emerge.

Use of the current minimum inhibitory and minimum bactericidal concentration (MIC/MBC) method in the clinic may need to be updated and/or complemented with newer techniques, and there is continued debate over an optimal animal model for compound screening ongoing in the research community [87]. Nevertheless, new and more effective drug treatments are unlikely to eliminate NTM infection and disease in NTM-susceptible individuals who are frequently reinfected with clinical NTM. Vaccination has the potential to protect patients from primary exposure to clinical NTM and could be used as immunotherapy treatment in combination with standard drugs. Furthermore, vaccines may represent the only realistic way of preventing the significant issue of reinfection. The call to action now lies with the urgent need to stimulate development of and advance effective vaccines and targeted immunotherapy against NTM.
